# Oral vitamin D supplementation at five times the recommended allowance marginally affects serum 25-hydroxyvitamin D concentrations in dogs

**DOI:** 10.1017/jns.2016.23

**Published:** 2016-07-29

**Authors:** Lauren R. Young, Robert C. Backus

**Affiliations:** Department of Veterinary Medicine and Surgery, College of Veterinary Medicine, University of Missouri, Columbia, MO 65211, USA

**Keywords:** Cholecalciferol, 25-Hydroxyergocalciferol, 24R, 25-dihydroxycholecalciferol, Parathyroid hormone, Ionised calcium, 1,25(OH)_2_D_3_, 1,25-dihydroxyvitamin D_3_, 24R,25(OH)_2_D_3_, 24R,25-dihydroxyvitamin D_3_, 25(OH)D, 25-hydroxyvitamin D, 25(OH)D_2_, 25-hydroxyergocalciferol, 25(OH)D_3_, 25-hydroxycholecalciferol, BW, body weight, CLIA, chemiluminesence immunoassay, iCa, ionised Ca, NRC, National Research Council, PTH, parathyroid hormone

## Abstract

Little is known regarding optimal vitamin D status in adult dogs. To date no studies on vitamin D supplementation for improving vitamin D status have been reported for adult dogs. The aims of this study were to identify dogs with low vitamin D status and evaluate an oral dosage of cholecalciferol (D_3_) for effectiveness in increasing vitamin D status. For this, forty-six privately owned dogs were evaluated. Of the dogs, thirty-three (or 71·7 %) had serum 25-hydroxyvitamin D (25(OH)D) concentrations less than 100 ng/ml, a minimum previously suggested for vitamin D sufficiency in dogs. Subsequently, thirteen dogs were enrolled in a supplementation trial. Dogs were given either a D_3_ supplement (*n* 7; 2·3 µg/kg^0·75^) or olive oil placebo (*n* 6) daily with food. Serum concentrations of 25(OH)D were determined at weeks 1, 3 and 6, and at the trial end. Only at the trial end (weeks 9–10) was 25(OH)D significantly greater (*P* = 0·05) in supplemented *v.* placebo dogs. Serum concentrations of 24R,25-dihydroxycholecalciferol determined at the trial end were about 40 % of that of 25(OH)D_3_ and not significantly different between the groups. Concentrations of parathyroid hormone, ionised Ca, P and creatinine measured in initial and final serum samples indicated supplementation caused no toxicity. We conclude that vitamin D_3_ supplementation at a dosage near the National Research Council recommended safe-upper limit was not effective for rapidly raising serum 25(OH)D concentrations in healthy, adult dogs. Further work is needed in evaluating the metabolism of orally administered D_3_ in dogs before dosing recommendations can be made.

Vitamin D has been extensively studied in human medicine since the discovery of its essentiality in the development and maintenance of a normal skeleton in the early 1900s. It is now known that through the interaction of vitamin D receptors in over forty tissues in the body, effects of 1,25-dihydroxyvitamin D_3_ (1,25(OH)_2_D_3_) go beyond Ca homeostasis and the prevention of rickets and osteomalacia to involve almost all body systems^(^^1^^)^.

It is widely accepted that the best indicator of vitamin D status is serum 25-hydroxyvitamin D (25(OH)D), as it is the most abundant circulating metabolite of vitamin D, and its concentration is determined by vitamin D intake^(^^2^^)^. Numerous reports have defined sufficient vitamin D status in people by evaluating the well-established inverse relationship between serum concentrations of 25(OH)D and parathyroid hormone (PTH)^(^^3^^–^^6^^)^. The 25(OH)D concentration at the inflection point of PTH, beyond which little further decrease in PTH is observed, has been interpreted as indicative of optimal Ca homeostasis and a marker of vitamin D sufficiency in people. However, this has led to some controversy in the definition of sufficient vitamin D status in people, as estimates of 25(OH)D concentrations necessary to suppress PTH concentrations have been variable^(^^7^^)^.

Recently the Institute of Medicine has recommended vitamin D deficiency in people be defined as 25(OH)D concentrations less than 20 ng/ml and insufficiency defined as 21–29 ng/ml^(^^8^^)^. While concentrations that are defined as insufficient are adequate for the prevention of bone disease, a growing body of evidence exists that concentrations greater than 30 ng/ml and closer to 40 ng/ml are desirable for optimal health and the prevention of chronic disease in people^(^^9^^)^. However, estimates are that 20–100 % of US, Canadian and European elderly men and women are vitamin D deficient, and current recommended allowances for vitamin D are unlikely to achieve sufficient vitamin D status for most people^(^^8^^,^^9^^)^.

Despite the vast research on vitamin D and health in people, no studies had attempted to define vitamin D sufficiency in adult dogs until recently. Comparing the relationship between 25(OH)D concentrations and intact PTH (iPTH), similar to human studies, investigators found that the median and variance in iPTH observations among dogs declined to a plateau when 25(OH)D concentrations were at 100 ng/ml^(^^10^^)^. A similarly significant drop in variability of mean serum canine C-reactive protein concentrations, a marker of chronic inflammation, was observed to occur at 25(OH)D concentrations of 100–120 ng/ml. Additionally, this study showed a decrease in the relative risk of developing cancers as 25(OH)D concentrations increased. While we believe these findings should be reproduced, this information provides novel evidence on which to base evaluation of vitamin D sufficiency in adult dogs for the prevention of chronic disease. However, reports of serum 25(OH)D concentrations amongst apparently healthy dogs widely differ^(^^10^^–^^13^^)^, with many that would be considered to have insufficient vitamin D status, based on this definition.

Unlike humans and several other species, dogs are unable to endogenously synthesise vitamin D_3_ in their skin in response to UV light^(^^14^^)^. Therefore, dogs are reliant upon their diet to supply their vitamin D requirements, primarily from the intake of vitamin D_3_ (cholecalciferol), but also vitamin D_2_ (ergocalciferol). The dietary vitamin D requirement for adult dogs has not been clearly established. The current recommended allowance by the National Research Council (NRC) is based on findings of studies identifying dietary concentrations of vitamin D that prevent skeletal abnormalities in puppies. In the absence of long-term studies in adult and pregnant and lactating dogs, dietary recommendations made for puppies are extrapolated to all life stages^(^^15^^)^, which may not be reflective of intake needed to achieve a specific health outcome.

In veterinary medicine, many investigators have reported associations between low serum 25(OH)D concentrations and canine mast cell tumour^(^^16^^)^, chronic kidney disease^(^^17^^)^, congestive heart failure^(^^18^^)^, inflammatory bowel disease^(^^19^^)^ and cancer^(^^10^^)^. Although cause and effect has not been established, these studies provide a basis for which vitamin D supplementation has the potential to improve health status, decrease disease risk, and be used as adjunct therapy in many diseases of dogs. To date, no published studies have reported on the effectiveness of oral vitamin D supplementation for increasing serum 25(OH)D concentration in dogs. Therefore, the aims of our study were to identify dogs with low vitamin D status as previously defined^(^^10^^)^ and evaluate responsiveness to vitamin D_3_ supplementation. Our hypothesis was that vitamin D_3_ supplementation at a daily dosage that is five times the NRC recommended allowance, but within the margins of the NRC safe upper limit, would effectively and efficiently raise vitamin D status without causing biochemical disturbances consistent with vitamin D toxicity when given to dogs with low 25(OH)D concentrations.

## Materials and methods

### Animals: vitamin D status survey

Following solicitation on our university campus announcement list-serve, forty-six privately owned, adult dogs aged 1·25–12·0 years (median 5·0 years) were volunteered for evaluation of vitamin D status. Various breeds were represented with sex distribution being thirteen male neutered (MN), thirty-two female spayed (FS) and one female intact. Body weights (BW) ranged from 5·3 to 49·0 kg (median 22·5 kg) with body condition scores of 4–8/9 (median 5/9). All dogs were reported by their owners as clinically healthy at the time of evaluation. Excluded were dogs weighing less than 2·2 kg, clinically ill dogs, those currently on vitamin supplements or medications that may alter Ca, P or vitamin D homeostasis or those on apparently nutritionally unbalanced diets. Owners completed a questionnaire regarding diet, environment, and current medications and medical conditions. Owners signed an informed consent form before participation which included the possibility of entry into a subsequent vitamin D supplementation trial if their dog was found to have low vitamin D status. The consent form and animal use protocol were reviewed and approved by our institution's animal care and use committee.

### Study design

BW and body and muscle condition scores were recorded for each dog at the time of blood collection. Approximately 5 ml of blood were collected from either the jugular or cephalic vein following an overnight food withholding. Extracted serum was stored at −20°C for later analysis of 25(OH)D concentration.

### Animals: vitamin D supplementation trial

Owners of thirteen dogs (four MN, nine FS) that were found to have low vitamin D status in the survey accepted participation in a clinical trial to evaluate the efficacy of oral vitamin D_3_ supplementation to improve vitamin D status. Signalment and other information for the groups are given in Table 1. Most of the dogs were housed primarily indoor with one dog both indoor and outdoor and one outdoor only. Complete diet histories were available for twelve of the thirteen dogs, of which eleven different commercially available dry diets were fed. The diets were labelled to indicate having undergone animal feed testing (*n* 8) or formulation (*n* 3) to meet Association of American Feed Control Officials (AAFCO) dog food nutrient profiles for adult maintenance (*n* 9) or all life stages (*n* 2). The vitamin D content of these diets was obtained by contacting the manufacturing company for a typical analysis of the final product, which all companies provided on an energy and as-is basis. Total daily vitamin D intake was calculated for twelve dogs by considering the amount of vitamin D present in the diet as provided by the manufacturers, food intake history from owners, and the dog's metabolic BW. The remaining dog was fed *ad libitum*, and the specific formulation of diet varied at times according to the owner, therefore obtaining dietary vitamin D content from the manufacturer or estimating dietary vitamin D intake was not possible. One diet fed to two dogs belonging to the same household did not meet AAFCO minimum vitamin D requirements on an energy basis. Based on food intake information provided by the owner, these dogs were not consuming enough diet to meet the NRC adequate intake of vitamin D on a metabolic BW basis (0·36 µg/kg^0·75^) (Table 2).

**Table 1.** Signalment and body condition data for dogs in the control (*n* 6) and treatment (*n* 7) groups of the vitamin D supplementation trial (Medians and ranges)

	Group			
	Control		Treatment	
	Median	Range	Median	Range
Body weight (kg)	31	13–49	28	13–39
Body condition score (1–9/9)	5	5–7	5	4–6
Age (years)	6·5	3·5–12·0	5·0	3·0–11·0
Breeds	Rottweiler (1), golden retriever (2), beagle (1), Australian shepherd (1), mixed breed (1)		Labrador retriever (2), golden retriever (1), bloodhound (1), Boston terrier (1), Weimaraner (1), mixed breed (1)

**Table 2.** Vitamin D content of diets fed and estimation of vitamin D intake from diet and supplement for dogs with complete owner-provided diet histories (Medians and ranges)

	Group			
	Control		Treatment†	
	Median	Range	Median	Range
Dogs (*n*)	5		7	
Intake (kJ/d)	4420	2630–6700	4140	1540–7350
Vitamin D (μg/418·4 kJ/diet)	1·9	0·6–2·6	0·5	0·3–2·0
Vitamin D intake from diet (μg/kg BW^0·75^)	1·9	0·6–2·3	0·4	0·2–1·8*
Vitamin D intake from supplement (μg/kg BW^0·75^)	0		2·3	2·2–2·3‡

BW, body weight.

* Values were significantly different between control and treatment groups (*P* = 0·03; Wilcoxon two-sample test).

† Daily oral vitamin D_3_ supplementation, 2·3 µg/kg^0·75^.

‡ Value based on measurement of vitamin D in supplement at the beginning of the trial.

### Study design

Immediately prior to entry into the trial, jugular or cephalic blood was again obtained following an overnight food withholding. A complete blood count with manual differential and clinical serum chemistry panel was obtained to screen each dog for underlying disease and to determine baseline values. Serum was stored at either −20 or −80°C in plastic tubes for later determinations of PTH and ionised Ca (iCa). The dogs were assigned to a control (*n* 6) or treatment group (*n* 7) based on the need for balancing for size of the groups, as well as age, sex and BW. Dogs belonging to the same household were assigned to the same group. Owners were blinded as to which group their dog was assigned.

Dogs of the treatment group received a vitamin D_3_ supplement in olive oil. The supplement was made by dissolving 29 mg vitamin D_3_ (cholecalciferol; Sigma-Aldrich) in 1·26 ml of ethanol and mixing 1 ml of the solution into 900 g of purified olive oil (Great Value; Wal-Mart Stores) to yield a concentration of 23 mg/l or 23 µg of vitamin D_3_/ml. The contents of one capsule of vitamin E (vitamin E supplement 1000 IU, Spring Valley; Wal-Mart Stores) were added to prevent oxidation of polyunsaturated fat (PUFA) in the oil during storage (1 mg dl-α-tocopherol/1 g of PUFA). The control group received a placebo of the same olive oil, prepared in the same manner with vitamin E. Once prepared, the supplement and control oil stock was stored in a dark cabinet at room temperature. Treatment and control dogs were dosed based on metabolic BW (0·1 ml/kg BW^0·75^ per d). At this dosage, treatment dogs received approximately 2·3 µg vitamin D_3_/kg BW^0·75^ per d, an amount that is 5·1 times the recommended allowance but does not exceed the safe upper limit recommended by the NRC for maintenance of adult dogs (2·6 µg/kg BW^0·75^)^(^^20^^)^. Owners applied the daily dose to food and were instructed not to alter diet, environment and exercise schedule during the trial.

Dogs were re-evaluated after initiating treatment or placebo supplement at weeks 1, 3, 6, and at the trial end, weeks 9–10. At each evaluation, venous blood was collected following an overnight food withholding, and serum was harvested for total 25(OH)D analysis. At the end of the trial, additional serum was obtained for analysis of: 24R,25-dihydroxyvitamin D_3_ (24R,25(OH)_2_D_3_), 25-hydroxycholecalciferol (25(OH)D_3_) and 25-hydroxyergocalciferol (25(OH)D_2_), PTH, iCa, P and creatinine. Owner compliance was assessed by weighing the dog's supplement prior to dispensing and at the conclusion of the trial. Vitamin D_3_ concentration was analysed in retained treatment oil stock and treated supplement returned by owners.

### Laboratory analyses

The complete blood counts (Sysmex xT-2000i; Sysmex America, Inc.) and clinical serum chemistry analyses (Beckman AU 400e; Beckman Coulter, Inc.) were performed at the University of Missouri Veterinary Medical Diagnostic Laboratory (Columbia, MO, USA). Serum total 25(OH)D was evaluated by a commercial laboratory (Veterinary Diagnostics Institute, Simi Valley, CA, USA) using a validated 25(OH)D assay (LIAISON; DiaSorin, Inc.). The assay is a direct, competitive chemiluminesence immunoassay (CLIA) for the quantitative determination of 25(OH)D in serum. The assay has been validated for use in dogs with an intra- and inter-assay coefficient of variation of 4·0 and 3·4 %, respectively^(^^10^^)^.

Concentrations were determined for the vitamin D metabolites, 24R,25(OH)_2_D_3_, 25(OH)D_2_ and 25(OH)D_3_, in 0·5 ml aliquots of thawed serum using a modification of a HPLC method previously reported^(^^21^^)^. For this, serum samples (0·5 ml) were incubated overnight at about 4°C with internal standard (2000 disintegrations per min of 25-[26,27-^3^H]hydroxyvitamin D_3_) prior to their extraction. AUC of HPLC peaks of 25(OH)D_3_ and 25(OH)D_2_ (Cerilliant) increased linearly with increasing mass of injected standard over the range of concentrations found in serum samples. Mobile phase was collected during 2 min periods coinciding with elution times of standards, 25(OH)D_3_ and 24R,25(OH)_2_D_3_ (Santa Cruz Biotechnology). The 25(OH)D_3_ fraction was dried by centrifugal evaporation, reconstituted in 6 ml of scintillation cocktail, and counted for ^3^H decay (2000 CA Tri-Carb; Packard Instrument). The 24R,25(OH)_2_D_3_ HPLC fraction was spiked with internal standard (10 ng of 25(OH)D_3_), dried by centrifugal evaporation and reconstituted in 130 µl of 20:80 ethanol–hexanes, the mobile phase of a second HPLC method for quantifying 24R,25(OH)_2_D_3_.

For the second HPLC method, 100 µl of the reconstitute was injected into mobile phase flowing at 1·0 ml/min on a column (Capcell Pak NH2, UG80, 5 µm, 4·6 × 250 mm; Shiseido) at ambient temperature (21–23°C). The AUC of UV peaks (265 nm) of 24R,25(OH)_2_D_3_ and 25(OH)D_3_ standards increased linearly with increasing mass of injected standard over the range of concentrations found in serum samples.

Serum PTH was evaluated by a commercial laboratory (Veterinary Diagnostics Institute, Simi Valley, CA, USA) with a direct, two-site, sandwich-type CLIA. The assay sensitivity, specificity, intra- and inter-assay CV have been previously reported^(^^10^^)^. iCa analyses were performed at Michigan State University Diagnostic Center for Population and Animal Health, Lansing, MI, USA (Nova 8 Plus; Nova Biomedical).

### Analysis of vitamin D_3_ in supplement

The vitamin D_3_ content in olive oil supplement was determined by a modification of a previously described method^(^^22^^)^. For each determination, 1 ml of oil was extracted with 9 ml of methanol to which was added 25 µg of internal standard (Ergocalciferol; Supelco) dissolved in methanol. A portion of the extract (10 µl) was injected onto an HPLC column (2·0 × 250 mm^2^, Ultrasphere ODS 5 µm Beckman Instruments) equilibrated with mobile phase (82:13:5, acetonitrile–methanol–water, by vol.) flowing at 0·3 ml/min. Concentrations of vitamin D_3_ and internal standard were determined from AUC absorbance recorded at 265 nm. The CV of vitamin D_3_ concentrations determined in the vitamin D_3_-containing supplement at 25 µg/ml was 2·3 %. The analyses were all run in the same batch.

### 25-Hydroyxvitamin D_3_ storage stability trial

To determine optimal storage conditions for 25-hydroxyvitamin D_3_, its stability was assessed over a period of 15 weeks of freezing and 14 d of refrigeration. Pooled beagle serum (25 ml; Innovative Research) was used for these analyses, to which 25(OH)D_3_ (2·5 µg in 0·25 ml of methanol) was added. Aliquots of 0·5 ml of serum were stored in a refrigerator (4°C) and in −20 and −80°C freezers. On days 0, 2, 4, 7, 10 and 14, of refrigerator storage and weeks 1, 2, 3, 5, 7, 9, 12 and 15 of freezer storage, 25(OH)D_3_ concentration was determined in duplicate aliquots by the method described for the analysis of serum 25(OH)D_3_. For this trial, 25(OH)D_2_ (50 ng in 10 µl of methanol) was used as the internal standard in place of the ^3^H label.

### Statistical analysis

Statistical analyses were performed using proprietary software (Excel 2013, Microsoft; SAS® 9.3, SAS Institute, Inc.). Variable observations were considered normally distributed if calculated mean and median differed by less than 10 %, kurtosis and skew statistics were between 1 and –1, and stem–leaf plots of observations indicated few outliers. All variable observations were found to be normally distributed except PTH, iCa, vitamin D intake, and change in pre- to post-serum 25(OH)D concentration. Following log transformation, PTH observations were normally distributed. For normally distributed observations, the significance of differences in variable observations within and between control and treatment groups were determined with paired and two-sample *t* tests, respectively. For non-normally distributed observations, central tendency and variation were expressed as median and range and variable differences within and between groups were tested for significance with signed-rank and Wilcoxon two-sample tests, respectively. The significance of variable correlations was tested with Pearson correlation. *P* values ≤ 0·05 were considered significant.

Mean and standard deviation were calculated for the 25(OH)D concentrations observed among the thirteen participating dogs prior to entry into the supplementation trial. Using these values and group sizes of six and seven dogs for the control and treatment groups, respectively, it was estimated that at a power of β = 0·8, the mean difference in 25(OH)D concentrations between the groups would have to be greater than 25 % to reach significance at α = 0·05.

## Results

### Vitamin D survey

**Fig. 1.**
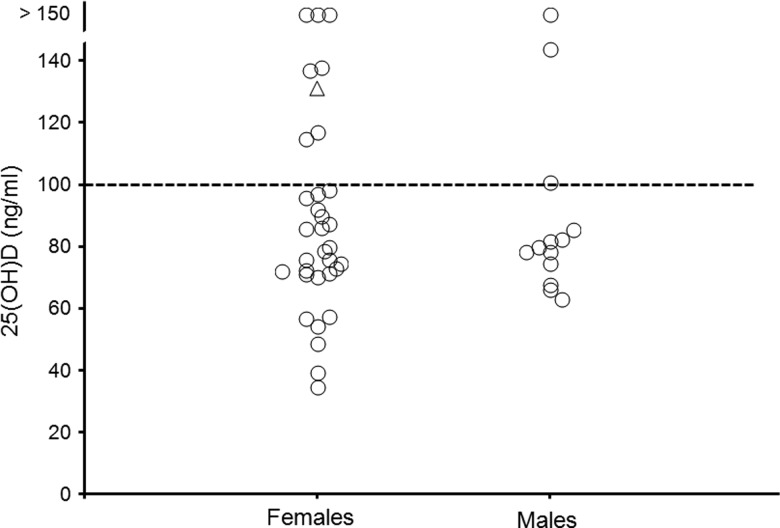
Serum total 25-hydroxyvitamin D (25(OH)D) concentrations of female (*n* 33) and male (*n* 13) adult dogs in vitamin D status survey. ∆, One intact female; ----, previously reported sufficient 25(OH)D status (100 ng/ml).

Serum total 25(OH)D concentrations ranged from 34·7 ng/ml to above the linear range of the total 25(OH)D assay, >150 ng/ml. We did not request dilution and re-assay for the few serum samples (*n* 4) with concentrations >150 ng/ml (Fig. 1). Thirty-three dogs (71·7 %) had 25(OH)D values below the previously reported minimum concentration indicating sufficient vitamin D status of 100 ng/ml^(^^10^^)^. The mean 25(OH)D values for females and males were 82·7 (sd  26·0) and 83·7 (sd 21·5) ng/ml, respectively, excluding the dogs with concentrations >150 ng/ml. The 25(OH)D concentrations were not significantly different between the two sexes.

### Vitamin D supplementation trial

**Fig. 2.**
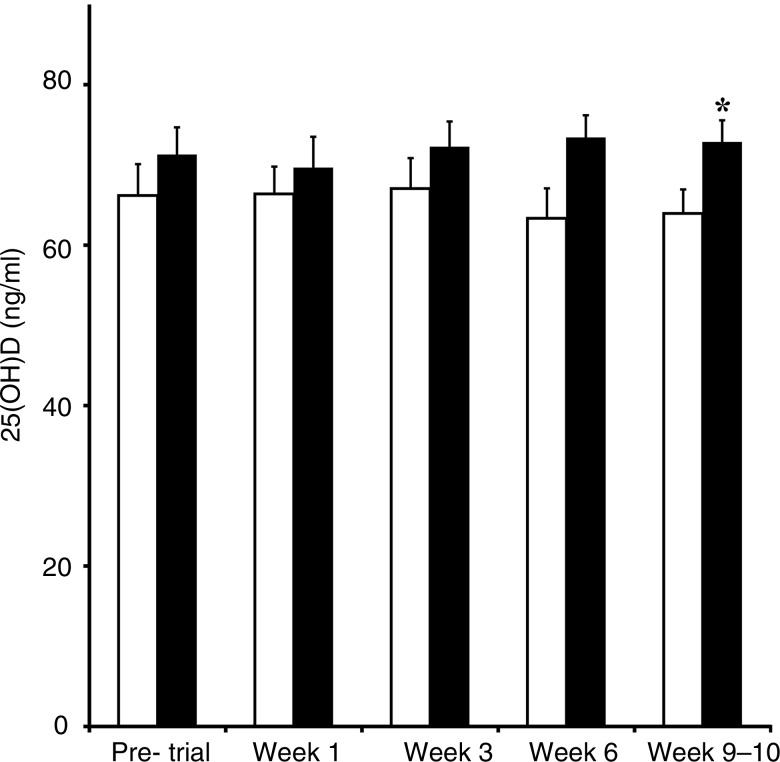
Serum total concentrations of 25-hydroxyvitamin D (25(OH)D) for control (□; *n* 6) and treatment (■; *n* 7) groups prior to beginning the vitamin D_3_ supplementation trial (pre-trial), and at weeks 1, 3, 6 and 9–10 after beginning supplementation. Values are means, with standard errors represented by vertical bars. * Mean value was significantly different from that of the control group (*P* = 0·05; two-sample *t* test).

The mean total 25(OH)D concentrations for the control group were 66·2 (sd 8·8) and 71·3 (sd 8·4) ng/ml in the treatment group prior to entry into the trial. Significant differences were not found within either the control or treatment group for 25(OH)D concentrations prior to supplementation and at weeks 1, 3, 6 and 9–10. In addition, no significant difference was found between the control and treatment groups prior to vitamin D_3_ supplementation, and at 1, 3 and 6 weeks later. Serum 25(OH)D concentrations were significantly greater (*P* = 0·05) in dogs in the treatment compared with the control group at weeks 9–10 (Fig. 2). However, analysis of the change in serum 25(OH)D concentrations from the beginning to end of the trial between the two groups did not reach significance. Initial and final serum 25(OH)D concentrations were not significantly correlated with initial and final serum iCa, PTH or P concentrations for either the control or the treatment group. While estimated dietary vitamin D intakes of the control group were significantly greater (*P* = 0·03) than those of the treatment group, initial serum 25(OH)D concentrations were not significantly correlated with initial vitamin D intake estimates (Table 2).

### Laboratory analyses

With only a few exceptions complete blood count and serum chemistry analyses results among the dogs were within the clinical laboratory reference ranges (Supplementary Table S1). Serum PTH, iCa, P and creatinine concentrations did not significantly change from the beginning to the end of the trial for either group. No significant difference was found for these variables between the control and treatment groups prior to and at the conclusion of the trial, except for iCa where the difference prior to entry into the trial was statistically significant between the groups (Table 3). Initial and final iCa concentrations were not significantly correlated with initial and final serum PTH concentrations for either the control or the treatment group.

**Table 3.** Serum biochemical analyses results for control (*n* 6) and treatment (*n* 7) groups prior to entry and at the conclusion of the vitamin D supplementation trial (Medians and ranges)

	Group							
	Control				Treatment†			
	Pre-trial		End-trial		Pre-trial		End-trial	
Variable	Median	Range	Median	Range	Median	Range	Median	Range
Intact PTH (pg/ml)	16	6–27·1	10·7	5·6–16·2	10·7	3·7–26·5	13·5	4·8–40·5
Ionised Ca (mmol/l)	1·3	1·1–1·3	1·3	1·3–1·4	1·3	1·3–1·4*	1·4	1·3–1·4
P (mmol/l)	1·3	1·1–1·4	1·3	1·1–1·5	1·4	1·2–1·7	1·3	1·1–1·6
Creatinine (μmol/l)	88·4	70·7–114·9	97·2	70·7–114·9	97·2	61·9–132·6	106·1	53·0–114·9

PTH, parathyroid hormone.

* Values were significantly different between pre-trial control and treatment groups (*P* = 0·02; Wilcoxon two-sample test).

† Daily oral vitamin D_3_ supplementation, 2·3 µg/kg^0·75^.

### Vitamin D metabolites

Extracts of serum samples used for quantification of vitamin D metabolites had clearly identifiable chromatographic peaks at retention times of standards for 25(OH)D_3_ and 24R,25(OH)_2_D_3_. A chromatographic peak coinciding with the retention time of 25(OH)D_2_ standard was not found in any extract. Calculated concentrations for serum 24R,25(OH)_2_D_3_ were about 40 % of those calculated for 25(OH)D_3_ (Table 4). They did not significantly correlate with the 25(OH)D_3_ concentrations. The mean 25(OH)D_3_ concentration of dogs of the treatment group was numerically greater than that of dogs of the control group, but this difference did not reach significance. Also, no significant difference was found between 24R,25(OH)_2_D_3_ concentrations of the two groups.

**Table 4.** Serum concentrations of vitamin D metabolites in control (*n* 6) and treatment (*n* 7) groups as measured by HPLC at the conclusion of the vitamin D supplementation trial (Mean values and standard deviations)

	Group			
	Control		Treatment*	
Vitamin D metabolite	Mean	sd	Mean	sd
25(OH)D_3_ (ng/ml)	40·4	9·9	47·8	6·4
25(OH)D_2_ (ng/ml)	ND		ND	
24R,25(OH)_2_D_3_ (ng/ml)	17·7	6	18·5	3·7

25(OH)D_3_, 25-hydroxyvitamin D_3_; 25(OH)D_2_, 25-hydroxyvitamin D_2_; ND, not detected (<1·0 ng/ml); 24R,25(OH)_2_D_3_, 24R, 25-dihydroxyvitamin D_3_.

* Daily oral vitamin D_3_ supplementation, 2·3 µg/kg^0·75^.

### Analysis of vitamin D_3_ in supplement

The concentration of vitamin D_3_ in the stock supplement quantified at the conclusion of the trial was measured to be 20·7 µg/ml, which was about 90 % of the initially targeted inclusion. Each treatment supplement containing vitamin D_3_ dispensed to owners was quantified at the conclusion of the trial. The vitamin D_3_ concentrations ranged from 19·1 to 22·4 µg/ml (median 19·7 µg/ml). The average measured concentration (19·9 µg/ml) represented a loss of 13 % of initial targeted inclusion (23 µg/ml).

### 25-Hydroyxvitamin D_3_ storage stability trial

The 25(OH)D_3_ concentrations in samples stored at 4°C for 2, 4, 7, 10 and 14 d were not significantly different from that determined on day 0. Likewise, the 25(OH)D_3_ concentrations in samples stored at −20 and −80°C for 1, 2, 3, 5, 7, 9, 12 and 15 weeks were not significantly different from that determined on day 0.

## Discussion

Our objective in evaluating the effectiveness of oral vitamin D supplementation to improve vitamin D status in apparently healthy adult dogs is based upon previous findings that dogs with chronic disease have lower vitamin D concentrations^(^^10^^,^^16^^–^^19^^)^. Similar to studies in human medicine, investigators of a recent study defined vitamin D sufficiency in a large cohort of adult dogs through the evaluation of biomarkers that are affected by vitamin D status^(^^10^^)^. The serum 25(OH)D concentration of dogs at an apparent asymptotic minimum of serum PTH concentration was found to be 100 ng/ml. To our knowledge this is the first published report defining sufficient vitamin D status in the adult dog in the maintenance state. While we believe that much investigation is needed to confirm this definition of vitamin D sufficiency, we also believe that research such as ours that evaluates a means to achieve a serum 25(OH)D concentration of 100 ng/ml is warranted.

Our finding that the majority (71·7 %) of apparently healthy dogs that we evaluated had serum 25(OH)D concentrations below 100 ng/ml (Fig. 1) is consistent with findings of recent studies^(^^10^^,^^13^^)^. The reason for this finding in dogs is unknown, but many factors including vitamin D inclusion differences in diets and individual variation in intake, absorption and metabolism of vitamin D are probably contributing. Unfortunately, the amount of vitamin D in the diet necessary to achieve a specific health outcome may be difficult to determine. One reason is that dietary vitamin D analyses may not account for all vitamin D activity. For example, adipose tissue and liver, which are common ingredients of dog foods, may contain substantial amounts of 25(OH)D relative to vitamin D^(^^23^^)^. Whereas the assayed vitamin D content of a dog food may be available upon request from a manufacturer; the 25(OH)D content of dog foods is not generally reported, and we suspect that it is not typically determined. Whether oral 25(OH)D has greater potency in raising vitamin D status in dogs is currently being investigated by the authors. In humans, ingested 25(OH)D has been shown to be many times more effective than vitamin D in raising serum 25(OH)D^(^^24^^)^.

Also problematic for determining sufficient dietary vitamin D inclusion for all dogs are factors evidenced to influence serum 25(OH)D concentrations in dogs, such as sex (males > females), reproductive status (intact > neutered) and breed^(^^13^^)^. The effect of reproductive status could not be confirmed with the current cohort studied, given that all but one dog was neutered. However, this may be one factor contributing to the low vitamin D status in the large majority of the dogs evaluated in this study.

Although serum 25(OH)D concentration is believed to indicate vitamin D status in dogs, it should be noted that methods by which 25(OH)D concentrations are assayed are not standardised, which can complicate the interpretation of results^(^^25^^–^^27^^)^. In our study, the CLIA method was used to measure total 25(OH)D concentrations, while a published HPLC method^(^^21^^)^ was used to quantify vitamin D metabolites not attainable through an immunoassay. We found that serum 25(OH)D concentrations determined by HPLC compared with those determined by the CLIA method used by the commercial laboratory were positively correlated but consistently lower (data not shown). This methodological difference was similar to that reported by Lensmeyer *et al*.^(^^21^^)^, the investigators from whom we adapted our HPLC methodology. Chromatographic methods, such as HPLC and LC/MS, compared with immunologically based methods like CLIA are touted as more accurate^(^^26^^)^.

The most significant finding in our study was only a modest difference (12 %) in vitamin D status when dogs are given an oral dosage of vitamin D that we believed to be substantial (Fig. 2). While a significant difference was found in serum 25(OH)D concentrations between the control and treatment groups at weeks 9–10, serum 25(OH)D concentrations of no dog in the treatment group increased to the reported sufficient vitamin D concentrations of 100 ng/ml. Therefore, our findings question the relevance of our vitamin D_3_ supplementation to achieve a specific health outcome in adult dogs. The reason our vitamin D supplementation was ineffective was not apparent; however, several possibilities to explain the results were investigated and are further discussed here.

To control for variables that have been reported to affect changes in serum 25(OH)D concentrations in people^(^^28^^,^^29^^)^, such as age, sex and BW, the control and treatment groups were balanced. These were not obese dogs on average, and the groups were of similar body conditions, so excess body fat, and therefore storage of vitamin D_3_ in adipose tissue, would not probably explain the weak response to vitamin D supplementation in our subjects.

The treatment schedule used in this supplementation trial followed that of a published human dose–response study that showed near-asymptotic serum 25(OH)D concentrations after approximately 10 weeks of daily oral administration of vitamin D_3_, irrespective of dose^(^^30^^)^. In this work on healthy human subjects, the greatest rise in serum 25(OH)D concentrations from baseline occurred within the first 3 weeks of supplementation. While it is unknown whether dogs respond to supplementation as humans do, it was anticipated that our study design would allow us to capture the early rise in 25(OH)D in response to supplementation. After finding a weak response in 25(OH)D concentrations of our treatment group, the trial was ended at 10 weeks, given we were unlikely to have further significant results maintaining this dose between the two groups in this trial.

In order to evaluate a practical means to vitamin D supplementation in dogs, an oral and voluntary route of administration was chosen in favour of intramuscular or intravenous routes. As a fat-soluble vitamin, D requires the presence of fat in the diet for adequate absorption in the animal. It is reasonable to believe the provision of vitamin D in an oil vehicle, as is typically used in human vitamin D supplements, and given with a meal would be absorbed in the healthy dog. As vitamin D dosing studies have not been reported in adult dogs, specifically for the intention of improving vitamin D status, the dosage of vitamin D supplementation chosen was kept just below the NRC recommended safe upper limit. To our knowledge, a minimum toxic oral dose of vitamin D has not been reported for adult dogs, so the NRC recommendation was heeded. Dietary vitamin D toxicity resulted in clinical signs and biochemical disturbances in two adult dogs when accidental over-supplementation was approximately twenty-nine times the safe upper limit^(^^12^^)^. A study in puppies fed a dietary concentration of approximately seventeen times the safe upper limit also did not result in clinical signs of vitamin D toxicity, but severely disturbed endochondral ossification^(^^31^^)^. Therefore, the dose of vitamin D_3_ supplement given, while not inconsequential, was not expected to result in toxicity.

Lacking receipt of vitamin D supplement by dogs and a decline in strength of supplement were not believed to be contributing factors in our results. Owner compliance was assessed by weighing the supplement prior to dispensing and at the end of the trial, as an indication that the supplement was being given in adequate amounts. Owners did not report problems with lack of intake by the dogs when the supplement in oil was applied on food. In addition, the retention of vitamin D_3_ in the supplements dispensed to owners as measured at the conclusion of the trial was still near the safe upper limit for vitamin D according to the NRC.

Serum concentrations of 24R,25(OH)_2_D_3_ in dogs have been reported to be higher than found in humans^(^^32^^)^ and other domestic species^(^^33^^)^. Investigation into the possible metabolic conversion of 25(OH)D by the dogs to 24R,25(OH)_2_D_3_ was conducted at the conclusion of the supplementation trial. As a major metabolite of 25(OH)D, 24R,25(OH)_2_D_3_ is produced in dogs by 24-hydroxlyase in the kidney and intestine^(^^34^^)^. Concentration of 24R,25(OH)_2_D_3_ in serum is well-established to positively correlate with serum 25(OH)D concentration^(^^31^^,^^35^^)^. This correlation has been documented in a study of puppies that reported that serum 24R,25(OH)_2_D_3_ concentrations were significantly higher in a treatment group consuming eight times the dietary vitamin D as the control group^(^^35^^)^. This difference in 24R,25(OH)_2_D_3_ concentrations was found after 6 weeks of supplementation, whereas 25(OH)D concentrations did not differ between the groups during the trial. In the present study, serum concentrations of 24R,25(OH)_2_D_3_ of the treatment group dogs were not significantly different from those of the control group dogs (Table 4). Therefore, it is reasonable to conclude that an increased metabolic loss of 25(OH)D to 24R,25(OH)_2_D_3_ does not explain the weak response that occurred in our treatment group to vitamin D supplementation.

A plausible explanation for the weak serum 25(OH)D response to our vitamin D supplementation might be low bioavailability of the supplement. Studies on factors affecting vitamin D absorption by dogs, distribution and metabolism of absorbed vitamin D are needed. Reports of supplement vehicle effects on oral vitamin D bioavailability are sparse^(^^36^^)^. To our knowledge vitamin D bioavailability comparisons between supplementation and dietary inclusion are lacking for dogs. Vitamin D infused into the portal blood of dogs is efficiently removed by the liver^(^^37^^)^. Therefore, a poor response in serum 25(OH)D to oral vitamin D supplementation in dogs could reflect extrahepatic uptake of vitamin D, such as into adipose tissue, and/or altered 25-hydroxlyation of vitamin D by the liver. Also, within the liver of dogs, 25(OH)D concentrations are reported to be as great as those of vitamin D^(^^38^^)^, and rate of 25-hydroxylation of vitamin D can be experimentally varied^(^^39^^)^.

A limitation of the present study is the small sample size. While our study design was of sufficient power to show that by the trial end (weeks 9–10) dogs receiving the vitamin D_3_ supplement had higher 25(OH)D concentrations as compared with control dogs, no dog in the treatment group reached the reported 25(OH)D concentration indicative of sufficiency. In addition, the inverse relationship between PTH and serum 25(OH)D concentrations that has previously been found in dogs was not evident in the present study. This was probably a result of large between-individual variance in PTH with any given serum 25(OH)D concentration in combination with our small group sizes not revealing the correlation.

In conclusion, our findings support those of others in that apparently healthy adult dogs vary significantly in their serum concentration of 25(OH)D, the indicator of vitamin D status. We performed a prospective trial in dogs with 25(OH)D concentrations below 100 ng/ml to assess the effectiveness of an oral dosage of vitamin D to improve their vitamin D status. We found a lesser response than anticipated at a daily dose of approximately 2·3 µg/kg BW^0·75^, which is substantially more vitamin D_3_ than the amount provided by these dogs’ commercially available diets. In light of the fact that this is the first study of its kind to report these findings in dogs, further investigation must be done before dosing recommendations can be made for dogs with low vitamin D status.

## Supplementary material

For supplementary material accompanying this paper visit http://dx.doi.org/10.1017/jns.2016.23. click here to view supplementary material
