# Supplementary material for: Oral vitamin D supplementation at five times the recommended allowance marginally affects serum 25-hydroxyvitamin D concentrations in dogs
Source: J Nutr Sci. 2016 Jul 29;5:e31. doi: 10.1017/jns.2016.23 (PMC4976120; doi:10.1017/jns.2016.23)
Supplement: Supplementary file 1 [file S2048679016000239sup001.docx]

**Supplementary Table S1.** Variables of serum complete blood count and biochemical analyses for all dogs prior to entry into the vitamin D supplementation trial (*n* 13)

| Variable | Median | Range | Reference range |
| --- | --- | --- | --- |
| WBC (x10^3/µL) | 8.22 | 6.94-12.47 | 4.53-14.99 |
| RBC (x10^6/µL) | 7.36 | 6.65-8.15 | 5.34-8.5 |
| Hgb (g/dL) | 17.6 | 15.8-19.5 | 12.3-19.7 |
| Hct (%) | 49 | 44-54 | 37-57 |
| MCV (fL) | 67 | 63-74 | 59-76 |
| MCH (pg) | 23.7 | 22.5-24.4 | 20.7-25.6 |
| MCHC (g/dL) | 35.8 | 33.2-36.6 | 32-36.4 |
| Platelet (x10^3/µL) | 204 | 137-685 | 200-500 |
| Segmented Neutrophil | 4.72 | 3.34-7.86 | 2.27-10.14 |
| Band Neutrophil | 0 | 0 | 0-0.26 |
| Lymphocyte (x10^3/µL) | 2.47 | 1.39-5.11 | 0.76-4.23 |
| Monocyte (x10^3/µL) | 0.39 | 0.00-1.16 | 0.15-1.35 |
| Eosinophil (x10^3/µL)  Basophil (x10^3/µL) | 0.62  0 | 0.28-1.38  0 | 0.08-1.1  No ref range |
| Reticulocyte count (%) | 0.7 | 0.2-1.2 | No ref range |
| Glucose (mg/dL)  Urea Nitrogen (mg/dL) | 83  16 | 63-101  10-21 | 81-133  8-28 |
| Creatinine (mg/dL) | 1 | 0.7-1.5 | 0.6-1.6 |
| Sodium (mEq/L) | 146 | 144-150 | 143-152 |
| Potassium (mEq/L) | 4.7 | 3.7-5.0 | 3.4-4.9 |
| Chloride (mEq/L) | 113 | 111-115 | 108-117 |
| Total CO_2_ (mEq/L) | 22 | 18-26 | 18-26 |
| Anion gap (mEq/L) | 16 | 14-17 | 13-22 |
| Albumin (g/dL)  Total protein (g/dL)  Globulin (g/dL)  Calcium (mg/dL) | 3.1  6.5  3.3  9.9 | 2.7-3.5  5.8-7  3.0-4.1  9.5-10.6 | 2.9-4.0  5.2-7.4  2.2-3.6  9.2-11.3 |
| Variable | Median | Range | Reference range |
| Phosphorus (mg/dL) | 4.2 | 3.4-5.3 | 2.0-5.0 |
| Cholesterol (mg/dL) | 221 | 164-282 | 133-338 |
| Total Bilirubin (mg/dL) | 0.2 | 0.1-0.3 | 0.1-0.4 |
| ALT (U/L) | 29 | 20-55 | 9-58 |
| ALP (U/L) | 29 | 9-90 | 5-129 |
| GGT (U/L) | 3 | 3-4 | 0-5 |
| CK (U/L) | 78 | 51-110 | 10-274 |
